# HTLV-1 modulates alternative splicing in CD4+ T cells in vivo

**DOI:** 10.1186/1742-4690-8-S1-A168

**Published:** 2011-06-06

**Authors:** Morgan Thenoz, Céline Vernin, Christiane Pinatel, Nicolas Nazaret, Joel Lachuer, Didier Auboeuf, Eric Wattel, Franck Mortreux

**Affiliations:** 1 Oncovirologie et Biotherapies, UMR5239 CNRS/ENS Lyon/UCBL/HCL, Hopital Pierre Benite, Lyon, France; 2 Oncovirologie et Biothérapies, Centre Léon Bérard, Lyon, France; 3 ProfileXpert, Neurobiotec Service, Bron, 69500, France; 4 Institut National de Santé et de Recherche Médicale U590, Centre Léon Bérard, Lyon, France

## 

Transcriptional dysregulation is the hallmark of HTLV-1 infected cells yet the impact of HTLV-1 on alternative splicing (AS) remains unknown. Given that about 95% of genes are spliced, we investigated the exon expression profiling of CD4+ T-cell clones obtained by limited-dilution cloning of PBMC deriving from HTLV-1 carriers. Overall, 12 infected clones clustering in infected, uninfected, PHA-stimulated or unstimulated CD4+ T cells were compared for exon RNA content using Exon Chip Human microarray. The splicing index method was used to identify differentially expressed exons in paired comparisons. The exon content of 558 genes differed between infected and uninfected CD4+ T cells. Of these, only 76 genes were found differentially expressed at the whole transcriptional level. 360 exon events (185/558 genes) could be manually annotated in FASTDB database and corresponded to exon skipping (59 genes), mutually exclusive exons (151), 5’ splice site (5’SS) (4), 3’SS (3), internal exon deletion (23), and intron retention (15). Some of these are already validated by exon specific RT-PCR experiments that are in progress. Exon-based hierarchical clustering analysis identified alternative exons associated with HTLV-1 infection and/or PHA stimulation. Ingenuity Pathway Analysis revealed new AS-based pathways of gene deregulation irrespective of whole transcriptional deregulation. These corresponded to hitherto unknown deregulation pattern of cell cycling and DNA damage response. Thus, HTLV-1 infection possesses a transcription-independent but AS-based signature that unmasks numerous new putative leukemogenic pathways.

